# Supplementary material for: Are carbon emissions trading and green financial instruments synergistic? -Comprehensive quantitative research based on content analysis
Source: PLoS One. 2024 Mar 7;19(3):e0298601. doi: 10.1371/journal.pone.0298601 (PMC10919601; doi:10.1371/journal.pone.0298601)
Supplement: S1 Data — (ZIP) [file pone.0298601.s001.zip › Data/地级市Z_F工作报告.zip!/╜¡╦╒/╤╬│╟╩╨/└┤╫╘╣1⁄2╓┌║┼í╛┬φ┐╦╩2╛▌═°í┐_2018.docx]

各位代表:

现在,我代表盐城市人民政府向大会作工作报告,请予审议,并请各位政协委员和其他列席人员提出意见。

2017年工作回顾

过去的一年,我们在省委、省政府和市委的正确领导下,认真贯彻落实党的十八大和十九大精神,坚持稳中求进工作总基调,坚定不移贯彻新发展理念,扎实推进产业强市、生态立市、富民兴市和“五个一”战略工程,全面做好稳增长、促改革、调结构、惠民生、防风险各项工作,较好地完成了市八届人大一次会议确定的主要目标任务。

一、经济发展稳中提质。全市经济稳定增长。经济总量首次突破5000亿元,预计地区生产总值达5050亿元,比上年增长6.8%,人均地区生产总值首次突破1万美元。一般公共预算收入360亿元,完成市人大常委会批准的调整指标。规模以上工业增加值2100亿元,企业增长面创近三年新高。固定资产投资4300亿元,增长11%;社会消费品零售总额1810亿元,增长11%。转型升级取得突破。三次产业比重为11:44.4:44.6,产业结构实现了从“二三一”到“三二一”的重大转变。新能源、节能环保、智能终端、大数据等产业发展势头强劲,战略性新兴产业产值增长19%,占工业产值比重提升1.5个百分点。新能源装机容量、发电量均列全省第一。实施千万元以上技改项目522项,机械、纺织、化工等传统支柱产业加快转型。扎实推进农业“接二连三”工程,龙头企业实现销售2500亿元,设施农(渔)业新增面积、“三品一标”农(渔)产品总数全省第一。加快建设31个市级服务业集聚区,旅游产业快速发展,服务业增加值、投资增幅、新增规模以上企业数均居全省前三。项目推进成效明显。举办一系列境内外重大招商活动,总投资超百亿元的中国电子盐城信息港项目、总投资超百亿美元的金光循环经济高科技产业园项目签约落户,盐都东山精密电子、东台远海科技等重大项目进展顺利。坚持每两个月举行一次现场观摩活动,持续推进“两重一实”项目建设,完成投资930.6亿元。鼓励企业“争星创优”,评定并表彰星级企业120家。科技金融支撑有力。全社会研发投入占GDP比重2.05%,净增高新技术企业81家,高新技术产业产值增长19%,发明专利申请、授权及有效发明专利拥有量增幅均列全省第一。金融业增加值占GDP比重首次超过5%,贷款总量苏北第一、增幅全省第三。

二、改革开放深入推进。各项改革取得新进展。不断深化供给侧结构性改革,全面完成去产能年度任务,全市商品住房库存去化周期缩短到7.6个月,新增直接融资435亿元,累计帮助企业降低成本60亿元。深化“放管服”改革,建成全市政务服务网络平台,推行“不见面审批”和数字化联合审图,实现“3550”改革目标。开展承担行政职能事业单位改革试点,稳步推进行政执法体制改革。投融资体制改革取得明显成效,设立并运行8支市政府产业基金,总规模超过230亿元,累计投放项目37个,完成投资88.1亿元,其中撬动社会资本67.8亿元。新水源地及跨区域引水工程项目成为财政部PPP示范项目。沿海开发实现新突破。港口能级进一步提升,滨海港区获批国家一类开放口岸,射阳港区获批临时开放口岸,大丰港区保税物流中心正式封关运营,响水港区保税物流园开工建设。全年港口货物吞吐量9500万吨,增长13%。临港产业快速发展,响水德龙镍业销售突破300亿元,滨海国电投协鑫2×100万千瓦火电机组并网发电,射阳远景能源等一批沿海重大项目投产达效。对外开放呈现新局面。中韩(盐城)产业园正式获得国务院批准,极大增强我市对外开放的差别化竞争力。北上海临港生态智造城全面开工,南北园区合作共建不断深化。开展园区“等级创建”,各类产业园区加快转型、提质增效。引进总投资5000万美元以上项目27个,实际利用外资增长10%。新批境外投资项目17个,外贸出口额增长24.7%,增幅全省第一。

三、生态环境更加宜居。生态保护力度持续加大。坚决落实中央环保督察整改要求,推动8个环境具体问题立行立改,饮用水源保护区、通榆河保护区、自然保护区、化工园区一批历史遗留问题得到有效解决。举办黄(渤)海湿地可持续发展与世界自然遗产国际研讨会,珍禽和麋鹿两个国家级自然保护区被列入世界自然遗产预备名录。通过国家卫生城市复审,荣获全省首批绿色建筑示范市。滨海、响水通过省级生态县验收,阜宁、大丰、东台经济开发区通过省级生态工业园区考核。“263”专项行动深入开展。关停化工生产企业32家,关闭燃煤锅炉583台,单位GDP能耗降幅全省第二,主要污染物削减量完成省定目标。开展大气污染专项治理行动,空气质量优良天数289天,全省最高,连续三年被省表彰为大气污染防治优秀城市。城乡环境综合整治成效明显。推行河长制,全面完成城乡河道整治三年任务。在市区部署开展“三治三化”专项行动,加强黑臭水体治理,完成1388幢楼宇亮化升级工程,新开工棚改安置住房4019套,基本建成3805套,整治9个老旧小区。农村环境不断改善,特色镇村培育取得新的进展,盐都大纵湖镇入选全国第二批特色小镇,市开发区汽车小镇、城南新区数梦小镇、大丰荷兰花海风情小镇、亭湖千鹤湾温泉风情小镇列入省级特色小镇创建培育名单,东台三仓镇兰址村等3个村庄成为省首批特色田园乡村建设试点。

四、群众生活持续改善。民生投入不断加大,全市财政民生支出590亿元,20件民生实事完成年度任务。城乡居民收入稳步增长。落实省富民33条和市富民10条,新增城镇就业12.1万人,城乡居民人均可支配收入分别增长8.3%和8.9%。完成12万建档立卡低收入人口和36个省定经济薄弱村脱贫任务。扎实推进健康扶贫工程,民生“三项保险”全面落实,惠及71.8万因病、因残、因灾祸导致的低保户及低保边缘群体。公共服务水平不断提升。新建、改扩建中小学、幼儿园61所,教育总投入达160亿元。引进合作办学项目34个、各类名师36人。扎实推进“健康盐城”建设,城乡居民均享有14类55项免费基本公共卫生服务,完成基层医疗卫生机构基础设施标准化建设三年目标,建立院士工作站3个、名院名医工作室33个,大丰家庭医生签约服务入选全国深化医改重大典型经验。覆盖城乡的公共文化服务体系基本形成,一批文艺精品力作获得省精神文明建设“五个一工程”奖和文华奖,荣获省首批“书香城市建设先进市”。广泛开展全民健身运动,成功承办第31届亚洲城市保龄球锦标赛。社会主义核心价值观深入人心,累计有61人(组)当选“中国好人”。社会保障体系更加完善。城乡基本社会保险覆盖率稳定在97%以上,29.5万低收入困难群体免费纳入城乡居民医保体系,在全国率先实现跨省异地就医直接联网结算。农村康居工程扎实推进,完成C级危房改造5.4万户。“6•23”灾后重建全面完成,总建筑面积近60万平方米的20个集中安置点如期交付,抗灾救灾工作取得决定性胜利。社会治理能力明显增强。法治盐城、平安盐城建设深入推进,“七五”普法广泛开展,系列“黄海行动”取得成效,刑事案件发案数全省最低,荣获全国社会治安综合治理优秀市。强化安全生产、应急管理、消防安全、食品药品监管、民族宗教事务等工作,安全生产事故起数和死亡人数保持“双下降”。开展“大走访大落实”活动,一批社会矛盾纠纷和信访积案得到化解,社会大局和谐稳定。支持服务国防和军队改革,促进军民融合发展,双拥创建成果更加巩固。

五、自身建设得到加强。全面增强“四个意识”。严守政治纪律和政治规矩,始终与以习近平同志为核心的党中央保持高度一致。不断提升政府工作组织化程度。开展“学先进、找差距,补短板、促发展”活动,提高工作标准,浓厚争先创优氛围。市财政局成为全国财政系统法治财政建设示范点,市环保局入围全国环境执法大练兵表现突出单位。深入推进法治政府建设。严格依法行政,推进政务公开。自觉接受市人大工作监督、法律监督和市政协民主监督,认真办理市人大代表建议197件、市政协委员提案285件。持续转变机关作风。严格执行中央“八项规定”和省、市委“十项规定”,落实政府系统党风廉政建设责任制,扎实抓好市委巡察反馈问题的整改落实。监察、审计、统计、物价和公积金管理等工作得到加强,档案、地方志工作取得新进展,妇女、儿童、青少年、老龄、残疾人、红十字、慈善事业、外事、台侨工作取得新进步。

各位代表,2017年我们深入贯彻落实党的十九大和省、市党代会精神,全力推进事关发展全局、事关重大民生的“五个一”战略工程。“一张网”快速推进。实施交通基础设施六大类39个项目,完成投资161亿元。内环高架快速路网三期工程开工建设,盐通高铁开工在即,盐徐高铁路基桥梁施工基本完成,盐连快铁盐城段主线基本贯通。宁盐等5条高速列入省新一轮高速公路网规划。提档升级农村公路2880公里,改造桥梁1823座。“一桶水”加快引进。新水源地及跨区域引水工程完成投资31亿元,占全部工程量的70%。盐龙湖水厂一期工程建成供水,通榆河取水口原水预处理净化、盐龙湖水库优化提升等工程快速实施。“一部车”难中求进。制定实施一揽子政策措施,促进汽车产业发展企稳向好,汽车产业规模达1600亿元。东风悦达起亚与百度合作打造国内合资品牌首款智能网联车,自主品牌纯电动车华骐300E成功上市。奥新新能源乘用车项目对外合作取得重大进展,与省产业研究院在盐城共同成立江苏新能源汽车研究院。“一片林”有力推进。大力实施沿海百万亩生态防护林工程,新造成片林12.4万亩,改造提升15.9万亩,新增城镇绿化3650公顷,造林总量全省第一,全市林木覆盖率达26.6%。建湖、阜宁创成国家园林城市。“一座城”品质提升。中心城市加快建设,实施150项城建重点项目,完成投资260亿元。“五大组团”扎实推进,南海未来城组团核心区启动建设,范公堤高铁综合枢纽组团高铁站房即将开工,大洋湾生态组团6000亩核心区基本成型,先锋岛生态组团启动环境整治及征收拆迁工作,斗龙港生态组团五大类17项重点工程全面动工。城市功能进一步完善,市体育中心荣获鲁班奖,国家智慧城市试点通过验收。扎实开展国家新型城镇化综合试点,县城及重点镇规划建设水平提高、承载能力增强,全市常住人口城镇化率达62.6%。

各位代表,过去一年的成绩来之不易,这是省委、省政府和市委正确领导的结果,是全市人民攻坚克难、团结奋斗的结果。在此,我谨代表市人民政府,向广大干部群众,向全体人大代表、政协委员,向离退休老同志,向各民主党派、工商联、人民团体和各界人士,向驻盐人民解放军、武警官兵、公安干警、省属驻盐各单位,向所有关心和支持盐城发展的海内外朋友,致以崇高的敬意和衷心的感谢!

在肯定成绩的同时,我们也清醒地看到,我市经济社会发展还存在一些突出问题:发展还不充分,实体经济水平有待提高,保持经济平稳增长的压力依然较大;转型升级步伐还不够快,创新驱动能力不强,经济发展的质量和效益还不够高;民生领域还有不少短板,城乡居民收入水平总体不高,脱贫攻坚任务繁重,基本公共服务保障能力还不强,环境保护压力较大;政府职能转变还不到位,机关作风和效能建设需要进一步加强,营商环境仍需优化,等等。对这些问题,我们一定会高度重视,采取有力措施切实加以解决。

2018年工作安排

2018年是贯彻党的十九大精神的开局之年,是决胜全面建成小康社会、实施“十三五”规划承上启下的关键一年。今年政府工作的总体要求是:全面贯彻党的十九大精神,以习近平新时代中国特色社会主义思想为指导,认真落实省委省政府和市委决策部署,坚持以人民为中心的发展思想,坚持稳中求进工作总基调,坚持新发展理念,以系统性思维推动高质量发展,深入推进“产业强市、生态立市、富民兴市”,持续实施“五个一”战略工程,着力构建大交通、大生态、大上海、大海洋、大开放的发展新格局,统筹做好稳增长、促改革、调结构、惠民生、防风险各项工作,加快建设“强富美高”新盐城,为如期全面建成小康社会打下坚实基础。

今年全市经济社会发展主要预期目标是:地区生产总值增长7%以上,一般公共预算收入增长6%左右,固定资产投资增长8%,居民消费价格涨幅控制在3%左右,城镇登记失业率控制在3%以内,全社会研发投入占地区生产总值比重2.13%,城乡居民收入分别增长8.2%和8.8%,节能减排完成省定目标。

重点抓好以下八个方面工作:

一、构建现代产业体系,推动实体经济更高质量发展

提档升级传统支柱产业。毫不动摇壮大“一部车”,聚焦新能源汽车、智能网联汽车、汽车后市场、自主品牌等领域,支持汽车产业做大做强。推动东风悦达起亚加大新车型投放和市场开拓力度,为其实现全年整车产销50万辆提供外部要素保障。支持华人运通创新团队加快产品研发制造,打造具有国际竞争力的自主品牌新能源汽车标杆企业。推进机械装备产业高端化发展,实施沈阳机床智造谷等一批重大项目。积极培育新医药产业,推动化工产业转型升级。开展纺织、食品等消费品行业增品种、提品质、创品牌专项行动,促进产业链重心向终端产品及高附加值环节攀升。扎实开展“产业项目载体提升年”活动,集中要素推动95个重大项目建设,实施500项投资超千万元技改项目,培育更多新的经济增长点。

培育壮大战略性新兴产业。大力实施“新能源+”,重点发展海上风电机组、高效太阳能电池组件,支持金风科技、上海电气、远景能源、阿特斯、天合国能、协鑫光伏等龙头企业发展,申办全球智慧能源大会,努力把新能源产业打造成为全市域地方标志性支柱产业。大力发展智能终端、大数据、节能环保、高端装备与新材料等新兴产业,推动东山精密电子、领胜科技、启航未来、阿里“数梦小镇”、华为大数据存储中心等重点项目建设,着力打造区域性特色产业。大力发展先进制造业,实施智能制造推进行动,重点培育5项示范引领项目和30项试点推广项目。启动“千企上云”计划,推动300家规模以上企业“上云”。

加快发展现代服务业。深入实施服务业发展三年行动计划,服务业增加值年增长8%以上。启动市级生产性服务业集聚示范区认定工作,实施服务业龙头企业培育行动,滚动培育30家重点企业。推进盐城金融智慧谷、中韩(盐城)产业园东方之洲、盐城高新区智创谷等服务业重点项目建设,带动金融、研发、电子商务等现代服务业加快发展。实施大洋湾生态旅游景区、丹顶鹤风情小镇、麋鹿风情小镇、黄海森林康养小镇等生态旅游重点项目,加快形成全域旅游发展新格局。

二、强化创新驱动,培育发展新动能

完善区域创新体系。创建国家创新型城市,构建以企业为主体、市场为导向、产学研相结合的科技创新体系。围绕产业链部署创新链,实施汽车、新能源、电子信息等主导产业发展三年行动计划,培育和引进一批掌握核心技术的龙头型创新企业,推动盐城俱罗OLED照明组件等一批重大储备项目尽快开工。围绕创新链完善资金链,加大财政扶持和金融支持力度,撬动社会资本共同建立新能源产业基金和智能制造产业基金。进一步深化产学研合作,支持企业对接高校科研院所,引进研发机构和先进实用技术,签约和实施产学研合作项目1000项以上。

增强企业创新能力。扎实推进千家高企培育计划,重点培育300家科技型企业,力争新增高新技术企业130家以上。创建国家知识产权示范城市,实施知识产权强企行动,推动100家以上企业建立知识产权管理标准化体系,发明专利申请突破7000件,实现高新技术企业发明专利申请全覆盖。实施企业创新能力提升计划,新创30家省级以上研发机构,争创省高新技术产品500只以上。打造一批重大成套新产品新技术示范应用工程,争创国家智能终端产业集群区域品牌建设试点。继续开展企业“争星创优”活动,营造企业家健康成长环境,弘扬优秀企业家精神,更好发挥企业家作用。

打造创新创业平台。鼓励重点园区建设特色产业创新平台,推进清华大学烟气多污染物控制技术与装备国家工程实验室等平台建设。探索离岸研发新模式,在上海设立盐城科创中心,实现科技研发在上海、成果转化在盐城。加强与国内外知名企业和科研院所合作,推动组建盐城智能制造创新中心,培育5家智能制造服务企业。落实聚力创新十条政策,加快发展科技金融和科技服务业,更大力度支持大众创业、万众创新。

加快集聚创新人才。持续推进“515”人才引进计划,更大规模招引紧缺人才和创新团队。实施新时代企业家英才培育工程和现代企业家能力提升工程,重点培养100名新生代企业家。建立人才柔性引进机制,更大力度从北京、上海、深圳等一线城市引进高精尖人才和海外高端人才。实施“盐城工匠”培育计划,组织“招工引匠”服务行动。支持在盐高校提升办学层次,推进对外合作办学,培养更多各类急需专业人才和高技能人才。

三、发展现代海洋经济,提升沿海开发水平

大力发展海洋产业。坚持陆海统筹、综合开发,做好“大海洋”文章。深入实施新一轮沿海开放开发行动计划,组织编制现代海洋经济功能区规划,建设沿海绿色产业带、城镇带、风光带和港口群,打造黄金海岸经济带,争创国家海洋经济示范区。推进总装机容量240万千瓦的8个海上风电场和一批新能源装备制造项目,打造国家级新能源产业基地。加快实施中海油LNG接收站等重大项目,提升临港能源储运能力。支持东台海洋工程特种装备产业园、大丰海洋生物产业园建设,引进海工装备、海洋生物制品、海洋生物材料、海洋创新药物研发及产业化项目,提升海水淡化成套装备产业化水平,建设江苏新型沿海先进制造业基地。充分利用沿海港口和盐田资源,推动金光循环经济高科技产业园等项目尽快开工,把滨海新区建设成为淮河生态经济带出海门户、国家河海联动开发示范区。放大两个国家级自然保护区品牌效应,按照世界自然遗产保护组织提出的“生态旅游”国际标准,打造湿地生态旅游目的地和国家滨海湿地特色旅游基地。扎实推进沿海百万亩现代渔业产业带建设,加快海洋渔业转型升级。

打造“飞地经济”示范样板。抢抓国家支持“飞地经济”发展政策机遇,探索建立“政府引导、企业参与、优势互补、园区共建、利益共享”的合作模式。依托307平方公里“上海飞地”做好“大上海”文章,推动沪苏大丰产业联动集聚区建设,加强与上海临港集团、光明集团战略合作,加快打造北上海临港生态智造城。依托17个南北共建园区,完善合作机制,创新合作方式,形成“飞地经济”发展新优势。

推进重大基础设施建设。启动实施基础设施建设三年行动计划,做好“大交通”文章。围绕构筑现代化综合交通“一张网”,推动高铁、高架、高速同步建设,实现互联互通。加快盐通高铁建设,建成盐徐高铁盐城段主体工程,盐连快铁全线通车,尽快融入上海“一小时经济圈”、北京“一日商务圈”,成为未来京沪高铁、京杭高铁、沪陕高铁三条国家高速铁路新干线的交汇点。积极做好大丰港区支线铁路前期工作。全面实施内环高架快速路网三期工程,年内完成主体工程50%以上。积极对接宁盐、东台至兴化、阜兴泰高速建湖至兴化段等高速公路项目,争取早日开工。推进一批国省道干线公路建设,建成一级公路100公里。实施盐城港扩容提升工程,推进四个港区深水航道、码头建设。新建一批水利、防洪、电力和信息化重大基础设施项目,更好地满足沿海经济发展需要。

四、推进农业农村现代化,促进乡村振兴

推动农村产业兴旺。围绕促进农村一二三产业融合发展,进一步调优农业结构,调减低效粮田70万亩,新增设施农业10万亩。继续实施“接二连三”工程,新培育60家以上农产品加工龙头企业。实施休闲农业与乡村旅游提升工程,开展创意休闲农业“百点创建”活动。大力发展“互联网+”农业,农业信息进村入户实现全覆盖,力争培育年交易额千万元以上的农业电商企业50家。坚持质量兴农、绿色兴农,培育一批农产品区域公共品牌,新增无公害、绿色、有机食品品牌200个。实施开放型农业发展三年行动计划,建设省级农产品出口示范基地,为扩大农产品出口创造更好条件。

促进城乡深度融合。进一步完善城乡统一的规划体系,优化镇村布局,深入实施国家新型城镇化综合试点,构建以6个县城、30个重点中心镇、200个新型农村社区为基本框架的新型城镇体系。引导人口有序流动,常住人口城镇化率提高到63.5%。因地制宜编制特色田园乡村规划,加快2个国家级、4个省级、14个市级特色小镇建设,建成20个省级美丽乡村。提升城乡一体化水平,完成农村公路提档升级三年行动计划,新改建农村公路1100公里、桥梁660座,新增10个镇开通镇村公交,推动基础设施、公共服务加快向乡村延伸。增强村级基层组织治理能力,加强乡风文明建设,促进乡村社会全面进步。

激发农村发展活力。巩固和完善农村基本经营制度,推进农村土地制度、产权制度改革,积极开展“三权”分置试点,稳妥开展农民承包地经营权抵押担保融资试点,因地制宜推进农村宅基地退宅还耕和有偿退出。在保持土地承包关系稳定长久不变的基础上,大力培育新型农业经营主体,发展多种形式土地适度规模经营,强化农业社会化服务,构建现代农业产业体系、生产体系、经营体系。

五、全面深化改革开放,增强发展动力

着力深化重点领域改革。以供给侧结构性改革为主线,重点在“破”“立”“降”上下功夫,大力破除无效供给,积极培育新动能,降低实体经济成本,推动经济发展质量变革、效率变革、动力变革。坚决去除违规产能、淘汰落后产能、取缔违法产能。加强住房制度改革和长效机制建设,加快建立多主体供应、多渠道保障、租购并举的住房制度,促进房地产市场平稳健康发展。推动融资平台市场化转型,加强政府性债务管理,强化金融监管,有效防范化解各类风险。进一步减少企业税费负担、制度性交易成本、用能成本、物流成本,确保为企业降低成本30亿元以上。推进企业上市,大力发展股权融资,扩大债券融资规模,提高直接融资比重。

进一步深化“放管服”改革,巩固“3550”改革成果,深化“不见面”审批,加快中介服务“网上超市”建设,推动全市政务信息系统整合共享。实施开发区大部门制机构改革,落实国家级开发区全链审批赋权。深化行政管理体制改革,推进综合行政执法体制改革和事业单位分类改革,用行政权力的减法换取市场活力的加法。统筹推进财税、金融、投融资、科技、文化、教育、卫生等领域改革,激发经济社会发展活力。

构建全面开放新格局。做好“大开放”文章,主动融入“一带一路”,在更大范围、更宽领域、更高层次上推进新一轮对外开放。加快中韩(盐城)产业园建设,全面落实中韩自贸协定,积极争取国家和省级层面政策支持,学习借鉴上海自贸区改革试点经验,努力把中韩(盐城)产业园建设成为深化供给侧结构性改革、加快建设创新型国家、推动形成全面开放新格局的示范区,中韩对接发展战略、共建“一带一路”、深化贸易和投资合作的先行区。积极推进我市与新万金等地的产业园区合作,重点发展新能源汽车、智能网联汽车和新一代信息技术产业,培育智能制造、光电、健康美容、文化创意、大数据、跨境电商等先导产业,打造中韩地方经济合作和高端产业合作新高地。

强化对外招商,在巩固对韩合作基础上,深化与欧美、港澳台经济合作,确保注册外资实际到账增长10%。创建省级出口产品质量安全示范区,提升汽车、机电、智能终端等高新技术产品的出口比重。依托盐城综保区和大丰港区、滨海港区、南洋机场三个国家一类开放口岸,推进跨境电商产业园、外贸综合服务中心等载体建设,培育对外贸易新业态。加强各类园区建设,探索完善园区“等级创建”,引导“一园一业”,推进园区特色化、差异化发展。

六、加大中心城市建设力度,着力提升功能品质

不断丰富城市内涵。围绕建美“一座城”,按照“框定总量、限定容量、盘活存量、做优增量、提高质量”的要求,统筹规划布局城市空间、规模、产业,推动城市发展从外延扩张向内涵提升转变。挖掘利用城市资源,加强城市风貌、公共空间、建筑形态、景观廊带等布局设计,体现盐城印记,让人们看得见历史、看得到文化、记得住乡愁。坚持老城改造与新城建设同步推进,实施一批城建重点项目,组织市区城市建设“家家到”观摩。创新城市治理方式,提升精细化管理水平。

大力推进“五大组团”建设。加快建设南海未来城核心区,实施一批配套工程,积极打造现代化、国际化、特色化新城区。高标准设计、高质量建设市区综合客运枢纽,推进站房建设,实现高铁、航空、高速、高架等多种交通方式无缝对接。加快建设大洋湾生态组团新一轮景观提升项目,实施水上乐园、盐渎古镇等一批重点工程。加大先锋岛组团建设力度,强化道路、绿化、管网等基础设施配套,持续提升老城区人居环境。全面拉开斗龙港生态组团框架,实施生态提升、旅游度假、文化教育、健康养生等工程建设,彰显郊野生态休闲旅游特色。

扎实开展“三治三化”专项行动。用三年时间,在市区开展水环境治理、棚户区治理、违法建设治理和城市绿化、亮化、美化“三治三化”专项行动,营造更加宜居宜业、共建共享的城市环境。全面治理水环境,年内整治黑臭水体河道15条,拆建新建防洪闸7座。全面治理棚户区,基本建成棚改安置住房1500套,完成棚户区改造征收1.68万户。全面治理违法建设,基本完成大市区违法建设专项治理任务,形成长效管控机制。全面实施城市绿化、亮化、美化工程,改善市容市貌、提升环境品质。新增城市绿地1400公顷,完成40个公园及街头绿地项目。实施文明素质提升工程,推动文明城市建设。

七、加强生态文明建设,致力打造美丽盐城

推进资源节约循环利用。坚持绿色发展,严把产业导向、项目准入、节能减排关口,设置能源消耗、水资源利用、土地开发利用上限,规范各类开发活动。建立工业企业资源集约利用综合评价机制,以单位用地税收、单位能耗税收、单位排放税收等为主要评价内容,引导企业科学合理利用要素资源,促进产业转型升级。推进工业废弃物综合利用示范试点,建设一批绿色制造重点项目,创建绿色制造体系。

坚决打好污染防治攻坚战。积极做好中央、省环保督察整改工作,扎实推进“263”专项行动。加强大气污染防治,确保空气质量优良天数比例78.5%以上。加强水污染防治,全面落实河长制,加快通榆河、蟒蛇河、射阳河、中山河等清水廊道建设,实施农村河道生态治理,完成县级水源地隐患整治任务。加强农业面源污染防治,实施农药化肥减量行动,持续开展畜禽养殖污染专项整治,全市规模畜禽养殖场治理率达70%。加强固体废弃物和垃圾处置,加快推进市静脉产业园、生活垃圾焚烧发电、建筑垃圾资源化利用及填埋、餐厨垃圾资源化利用等项目,确保城乡生活垃圾无害化处理率达92%。

强化生态保护建设。做好“大生态”文章,全面落实主体功能区规划,严守生态保护、永久基本农田、城镇开发边界“三条红线”。弘扬塞罕坝精神,大力实施“一片林”工程,高标准创建国家森林城市,新增成片造林15万亩、改造提升15万亩,建成森林小镇10个、森林村庄100个。实施湿地生态修复工程,建立盐城滨海湿地研究院,建设系统化的科学监测平台,提高湿地保护水平。加快创建生态保护特区,积极做好黄(渤)海湿地世界自然遗产申报工作。创新生态文明体制机制,探索建立国家公园,争创国家生态文明建设示范市。

八、切实保障改善民生,不断增进群众福祉

千方百计促进富民增收。加大省富民33条和市富民10条政策落实力度,坚持产业升级和扩大就业规模、调整就业结构联动推进,强化职业技能培训,突出抓好高校毕业生、退役军人等重点群体就业,全年新增城镇就业9万人。实施“创业盐城”行动计划,深入推进以大学生、在外成功人士等群体为重点的全民创业,支持小微创业者在“双创”中实现创业致富。落实最低工资标准调整政策,引导企业形成以一线职工为重点的工资正常增长机制。大力发展民宿旅游、农村电商等新产业、新业态,促进农民增收致富。

坚决打好脱贫攻坚战。持续精准发力,因村因户施策,开展产业扶贫、就业扶贫、健康扶贫、教育扶贫、兜底扶贫和南北镇村挂钩帮扶,确保12万低收入人口、35个省定经济薄弱村实现脱贫目标。推进产业扶贫,加快培育一批能带动低收入农户和经济薄弱村长期稳定增收的优势特色产业。推进就业扶贫,加强就业岗位配送,实施家门口就业工程。推进健康扶贫,实行建档立卡低收入人口基本医疗保险个人缴费部分全额补贴,着力解决因病因残致贫返贫问题。推进教育扶贫,加大建档立卡家庭子女教育资助和生活补助力度。推进兜底扶贫,将农村低保标准逐步提高到省定扶贫标准以上,加大临时救助力度,帮助低收入农户解决特殊困难。充分发挥“阳光扶贫”监管系统作用,压实扶贫工作责任,让扶贫全过程公开透明。扎实做好援疆、援青以及与陕西铜川、辽宁阜新对口合作工作,推动优势互补、互惠互利、共同发展。

提高公共服务保障水平。落实省“十三五”基层基本公共服务功能配置标准,着力构建功能齐全、资源共享、持续完善、全面覆盖的基本公共服务体系。坚持教育优先发展,统筹城乡教育资源配置,继续引进名校名师,推进义务教育优质均衡发展,着力解决中小学生课外负担重、“择校热”“大班额”等突出问题。全市新建、改扩建中小学、幼儿园51所,其中主城区新建8所。推进“健康盐城”建设,实施基层卫生服务能力提升三年行动计划,积极构建覆盖城乡居民的“15分钟健康服务圈”,加快市中医院南扩等工程建设,继续实施名院名医“双引”工程,提升医疗卫生服务水平。广泛开展全民健身运动,市区新建25公里健身步道,升级50个老旧小区体育设施。大力推进文化惠民,开展送文化到基层系列活动,创作更多文艺精品,丰富群众文化生活。实施统一的城乡居民医疗保险制度,推进各项社会保险扩面征缴提质增效。实施托底救助工程,推进残疾人之家建设,加快养老服务业发展,年内实现以农村敬老院为基础的乡镇养老服务中心全覆盖。

坚持尽力而为、量力而行,聚焦社会事业、社会保障、公共安全、生态环境等公共服务领域,继续实施20件民生实事,完成年度投资160亿元。加快新水源地及跨区域引水工程建设,确保6月底主线建成通水,让惠及500万人民的“一桶水”流向千家万户。推进农村康居工程,年内完成农村B级危旧房改造10.3万户。实施市区无主管老旧小区物业托底管理、路灯改造、公共安全技防等实事工程,不断增强群众获得感、幸福感、安全感。

打造共建共治共享的社会治理格局。深入推进平安盐城、法治盐城建设,构建立体化、信息化社会治安防控体系。进一步健全基层社会治理体系,增强社区服务能力,推进网格化管理全覆盖。严格落实安全生产责任制,扎实开展安全生产大检查大整治大落实专项行动,坚决防范重特大事故发生。完善食品药品安全监管机制,切实保障人民群众饮食、用药安全。健全社会矛盾多元化解机制,推进信访积案攻坚,促进社会大局和谐稳定。加强国防动员、人民防空、双拥共建和民兵预备役工作,积极推进军民融合深度发展,推动民族宗教、气象、防震减灾、地方志、红十字和关心下一代等事业再上新台阶。

全面深化政府自身建设

面对新时代社会主要矛盾的深刻变化,面对繁重艰巨的改革发展稳定任务,我们将始终坚持以人民为中心,不忘初心、牢记使命,勇于担当、砥砺前行,切实做到为人民服务、对人民负责、受人民监督、让人民满意。

一、旗帜鲜明讲政治。以政治建设为统领,切实增强“四个意识”。坚决维护以习近平同志为核心的党中央权威,严格遵守政治纪律和政治规矩。坚决服从党对一切工作的领导,按照中央和省、市委决策部署抓工作、办事情、谋发展。坚持用习近平新时代中国特色社会主义思想指导实践,扎实开展“不忘初心、牢记使命”主题教育,全面加强政府系统自身建设,打造一支讲政治、守规矩、素质高、本领强的公务员队伍。

二、忠诚履职善执行。弘扬专业精神,培养专业能力,提高工作执行力。在转变职能中提高执行力,加大简政放权力度,切实把政府该放的权放到位、该管的事管到位。在依法行政中提高执行力,推进重大行政决策合法性审查和公平竞争审查,规范行政行为。在调查研究中提高执行力,向基层学习、向群众学习、向专家学习,在实践中汲取基层经验,提升专业水平。在学习先进中提高执行力,继续开展“学先进、找差距,补短板、促发展”活动,推动政府工作部门攻坚克难、进位争先。在细化落实中提高执行力,对既定的工作部署制订时间表路线图,分解任务、落实责任,确保各项工作落地生效。

三、驰而不息转作风。突出问题导向,持续正风肃纪。坚决贯彻落实中央“八项规定”实施细则精神,严肃整治“四风”问题新表现。加大对群众身边不正之风的整治力度,坚决纠正不作为、乱作为、表态多调门高、行动少落实差等突出问题。大兴求真务实之风,沉下身子察民情解民忧、顺民意办实事,不搞形式主义,不做表面文章,走好新时代群众路线。以更实举措优化营商环境,持续深化“暖企行动”,构建“亲”“清”新型政商关系,让各类市场主体放心创业、安心发展,建设人民满意的服务型政府。

四、反腐倡廉树形象。严格“一岗双责”,落实党风廉政建设责任制。切实抓好市委巡察反馈意见整改落实,推动主体责任落到实处。自觉接受市人大法律监督、工作监督和市政协民主监督,高度重视审计监督、司法监督、社会监督和舆论监督,让权力在阳光下运行。坚持用制度管事,强化行政审批、公共资源交易、工程项目、政府采购等重点领域和关键环节风险管控,着力构建廉政风险防控长效机制。规范财政收支管理,严控“三公”经费,把有限的资金和资源更多用在促发展、保民生、补短板上,树立为民务实清廉的良好形象。

各位代表,新时代新征程已经开启,我们肩负的责任更加重大,使命更加光荣。让我们更加紧密地团结在以习近平同志为核心的党中央周围,在省委省政府和市委的坚强领导下,凝心聚力、真抓实干,锐意进取、攻坚克难,全面完成今年各项目标任务,为决胜全面建成小康社会、建设“强富美高”新盐城不懈奋斗!
